# Cycloid psychosis

**DOI:** 10.1192/j.eurpsy.2021.1702

**Published:** 2021-08-13

**Authors:** R. Hernandez Anton, L. Aranguren Conde, M. Basteguieta Gardeazábal, P. Antía Ozcariz, N. Cancelo Zariquiey, N. De Sousa Figueiredo, V. Fronda Salinas

**Affiliations:** PsiquiatrÍa, COMPLEJO HOSPITALARIO DE NAVARRA, PAMPLONA, Spain

**Keywords:** cycloid psychosis, Kretschmer, Bleuler, Schneider

## Abstract

**Introduction:**

When we talk about cycloid psychosis we have doubts about their nosological enclave; whether they should be considered as a subform of schizophrenia or as independent psychoses.Some solutions were proposed, such as the thesis of mixed psychoses (Kretschmer) or that of intermediate forms (Bleuler, Schneider). Cycloid psychoses and bouffée delirante are recognized in ICD-10 under the name of acute polymorphic disorder without symptoms of schizophrenia (F23.0) and with symptoms of schizophrenia (F23.1).

**Objectives:**

Clinical case

**Methods:**

We present the case of a 16-year-old patient with no psychiatric history, with medical background of epilepsy; she was in fllow-up by Neurology and in treatment with valproate.Neurology indicates to stop treatment; it is then whwn the patient begins to appear disoriented, confused, with significant anguish and lability and regressive behaviors.She has sudden mood swings (from laughing to crying); sudden changes in emotional reaction (from distress to anger) and sudden changes in behavior (from agitation to prostration); verbiage with pressure of speech and dysprosodia; delusional ideation and incongruous affect; visual, auditive and kinesthetic hallucinations with important repercussion. We request blood and urine tests, drug test, EEG, cranial MRI.

**Results:**

She presents fluctuating, polymorphic and unstable affective and psychotic symptoms. What is the most appropriate diagnosis? We treat the patient with antipsychotic, mood stabilizer and anxiolytic treatment.

**Conclusions:**

Psychopathology in early ages is not so clearly defined and it can take very different forms. The diagnosis of cycloid psychosis can be useful as well as necessary to describe certain patients with similar characteristics and different from other groups.

**Disclosure:**

No significant relationships.

